# Integration of ABA, GA, and light signaling in seed germination through the regulation of ABI5

**DOI:** 10.3389/fpls.2022.1000803

**Published:** 2022-08-24

**Authors:** Hongyun Zhao, Yamei Zhang, Yuan Zheng

**Affiliations:** ^1^State Key Laboratory of Crop Stress Adaptation and Improvement, School of Life Sciences, Henan University, Kaifeng, China; ^2^Sanya Institute of Henan University, Sanya, China

**Keywords:** abscisic acid, gibberellins, light signaling, seed germination, ABI5, Arabidopsis

## Abstract

Seed germination is precisely controlled by a variety of signals, among which light signals and the phytohormones abscisic acid (ABA) and gibberellin (GA) play crucial roles. New findings have greatly increased our understanding of the mechanisms by which these three signals regulate seed germination and the close connections between them. Although much work has been devoted to ABA, GA, and light signal interactions, there is still no systematic description of their combination, especially in seed germination. In this review, we integrate ABA, GA, and light signaling in seed germination through the direct and indirect regulation of ABSCISIC ACID INSENSITIVE5 (ABI5), the core transcription factor that represses seed germination in ABA signaling, into our current understanding of the regulatory mechanism of seed germination.

## Introduction

Seed germination is the first step in the growth of flowering plants, and its precise regulation is important for selecting appropriate conditions for seed development (Finch-Savage and Leubner-Metzger, 2006; Rajjou et al., 2012). Among the various factors that regulate seed germination, the phytohormones ABA and GA are considered to be the most important endogenous signals, and the balance between ABA and GA is considered to be the decisive factor in determining seed dormancy or germination (Shu et al., 2016; Vishal and Kumar, 2018). Among the external environmental factors, light is considered to be one of the key factors affecting seed germination (Kami et al., 2010; Li et al., 2011). Light-mediated regulation of seed germination involves the control of both GA and ABA signals (Kim et al., 2008; Gabriele et al., 2010). Although many articles have described the cooperation between ABA and GA (reviewed by Vishal and Kumar, 2018) or ABA and light signaling (reviewed by Bulgakov and Koren, 2022), there are no works focused on the network of ABA, GA, and light signaling, especially in seed germination, in which all three signals play crucial roles. Therefore, the analysis of the cooperation among these three signals is very important for us to fully understand the signal regulatory network of seed germination.

However, ABA, GA, and light signals have been widely studied, and a large number of regulatory factors have been discovered, so their integration has been difficult. The core transcription factor ABI5 in ABA signaling plays crucial roles in regulating seed germination, and its function and regulatory mechanisms have been extensively studied (reviewed by Yu et al., 2015; Skubacz et al., 2016; Collin et al., 2021). Multiple signals participate in ABA- and GA-mediated seed germination by regulating ABI5 and using ABI5 as a final repressor of seed germination (Hu et al., 2019; Ju et al., 2019; Zhao et al., 2019). In this paper, we selected ABI5 as the key for integrating ABA, GA, and light signals into our current understanding of the signal network of seed germination.

## ABI5: The crucial regulator of ABA-mediated seed germination inhibition

ABA is an important phytohormone that regulates numerous developmental processes and responses to the environment (Cutler et al., 2010; Chen et al., 2020). In recent decades, the core components of ABA signaling have been extensively studied. Fourteen START-domain-containing proteins, such as pyrabactin resistance1 (PYR1)/pyrabactin resistance1-like (PYL)/regulatory components of ABA receptor (RCAR), have been shown to function as ABA receptors. SNF1-related protein kinase 2s (SnRK2s) serve as the key positive regulators. Upon binding to ABA, PYR1/PYL/RCARs interact with type 2C protein phosphatases (PP2Cs) and form a stable complex, leading to the release of SnRK2s from PP2C-SnRK2 complexes (Cutler et al., 2010). Activated SnRK2s subsequently phosphorylate downstream transcription factors such as AREB/ABF and ABI5 to regulate the expression of ABA-responsive genes (Fujii et al., 2009; Vishwakarma et al., 2017).

The bZIP transcription factor ABI5 plays a critical role in ABA-mediated seed germination and postgermination growth inhibition (Finkelstein and Lynch, 2000). ABI5 mutation causes insensitivity to ABA-dependent seed germination and vegetative growth arrest, and overexpression of *ABI5* results in enhanced sensitivity to ABA. ABI5 directly binds to the ABA-responsive element (ABRE) within the promoters of target genes, such as *EARLY METHIONINE-LABELED 1* (*EM1*) and *EM6*, to activate their expression (Carles et al., 2002). SnRK2s activate the transcriptional activity of ABI5 through phosphorylation (Nakashima et al., 2009).

### Regulation of ABI5 at the transcript and translation levels in ABA signaling

High levels of ABI5 transcript and protein accumulate in seeds but sharply decline during seed germination. ABA induces high expression and protein accumulation of ABI5 to repress seed germination. Given the important role of ABI5 in ABA signaling, the regulation of ABI5 has been extensively studied (reviewed by Yu et al., 2015; Skubacz et al., 2016; Collin et al., 2021). At the transcriptional level, the AP2/ERF transcription factor ABI4 and MADS-Box transcription factor AGL21 positively regulate *ABI5* expression, while WRKY transcription factors, including WRKY40, WRKY18, and WRKY60, function as the main repressors of *ABI5* expression (Bossi et al., 2009; Liu et al., 2012; Yu et al., 2017). In addition, ABI5 can directly promote its own expression (Xu et al., 2014a).

At the protein level, ubiquitination and 26S proteasome-mediated protein degradation play a critical role in the regulation of ABI5 stability. ABI5 can shuttle between the cytosol and nucleus, and cytoplasmic degradation of ABI5 is mediated by the RING-type E3 ligase KEEP ON GOING (KEG). KEG directly interacts with and ubiquitinates ABI5 to maintain ABI5 protein at low levels. ABA enhances ABI5 stability by inducing the self-ubiquitination and proteasomal degradation of KEG (Liu and Stone, 2010). The stability of ABI5 in the nucleus is modulated by CUL4-based (CULLIN 4) E3 ligases. DWD HYPERSENSITIVE TO ABA 1/2 (DWA1/2) and ABD1, which serve as the substrate receptors for CUL4 E3 ligase, interact with ABI5 to promote the degradation of ABI5 (Lee et al., 2010; Seo et al., 2014). S-nitrosylation of ABI5 at Cys-153 facilitates its degradation through CULLIN4-based and KEG E3 ligases (Albertos et al., 2015). Sumoylation of ABI5 by SIZ1, a small ubiquitin-related modifier (SUMO) E3 ligase, protects ABI5 from degradation. However, sumoylation negatively regulates ABI5 activity and prevents the degradation of ABI5 through the ABI5 location in alternative nuclear bodies (Miura et al., 2009).

### Regulation of ABI5 by its binding proteins

In addition to diverse posttranslational modifications, cofactors also seem to be very important in regulating ABI5 function. ABI3 was first identified as an interaction partner that activates ABI5 to regulate the ABA response during seed germination (Nakamura et al., 2001). The VQ motif-containing transcriptional regulators VQ18 and VQ26 physically interact with ABI5. VQ18 and VQ26 negatively modulate the ABA response during seed germination *via* repression of ABI5 transcriptional activity (Pan et al., 2018). The Mediator subunits MED25 and MED16 have been found to interact with ABI5. The combination of MED25 with ABI5 prevents the binding of ABI5 to the promoter of ABA-responsive genes (Chen et al., 2012). MED16 acts as a positive regulator of the ABA response opposite MED25. MED16 competes with MED25 to interact with ABI5 to regulate the ABI5-mediated expression of ABA-responsive genes (Guo et al., 2021).

Factors involved in controlling ABI5 stability have also been identified. ABI five binding protein 1 (AFP1) attenuates ABA signals by interacting with ABI5 and promoting its proteasomal degradation (Lopez-Molina et al., 2003). The transducin/WD40 repeat-like superfamily protein XIW1 is a nucleocytoplasmic shuttling protein and *Arabidopsis* exportin 1 (XPO1), an importin-β-like nuclear transport receptor (NTR), mediates the nuclear export of XIW1. XIW1 mainly localizes to the cytoplasm under normal conditions, and ABA facilitates the nuclear retention of XIW1. XIW1 interacts with and maintains the stability of ABI5 in the nucleus, thus playing a positive role in ABA responses (Xu et al., 2019).

As the key regulator in the ABA-mediated inhibition of seed germination, ABI5 also acts as a connection point for other signals involved in the regulation of seed germination. The bHLH transcription factor INDUCER OF CBF EXPRESSION1 (ICE1), which plays a central role in the cold response, and BRINSENSITIVE1 (BRI1)-EMS-SUPPRESSOR1 (BES1), a key transcription factor in the BR signaling pathway, as well as JAZ proteins, pivotal repressors of the JA signal, were found to participate in ABA-mediated seed germination inhibition by directly interacting with ABI5 and repressing its transcriptional activity (Hu et al., 2019; Ju et al., 2019; Zhao et al., 2019).

## ABI5: The final common repressor of germination in response to ABA and GA

GA is a phytohormone that regulates plant development and participates in processes including seed germination, seedling growth, and flowering (Xu et al., 2014b; Wu et al., 2021). GA is another critical phytohormone that controls seed germination, and antagonism between GA and ABA is the decisive factor in regulating seed germination. Exogenous application of GA can help seeds break dormancy, while application of the GA synthesis inhibitor paclobutrazol (PAC) can inhibit the germination of seeds (Shu et al., 2016). The GA-deficient mutant *gibberellic acid-requiring1* (*ga1*) is unable to germinate without exogenous GA (Sun and Kamiya, 1994). The GA receptor GID1 interacts with DELLA proteins, which act as key repressors in the GA signaling pathway. The combination of GA promotes GID1 to interact with SCF E3 ubiquitin ligase SLEEPY1 (SLY1), leading to the degradation of DELLA proteins, such as REPRESSOR OF ga1-3 (RGA) and RGA-like 2 (RGL2), and the release of GA signaling (Dill et al., 2004; Ueguchi-Tanaka et al., 2005; Davière and Achard, 2013, 2016; Claeys et al., 2014).

### DELLAs-ABI5 module: The hub that connects ABA and GA signaling in seed germination

**Figure 1:**
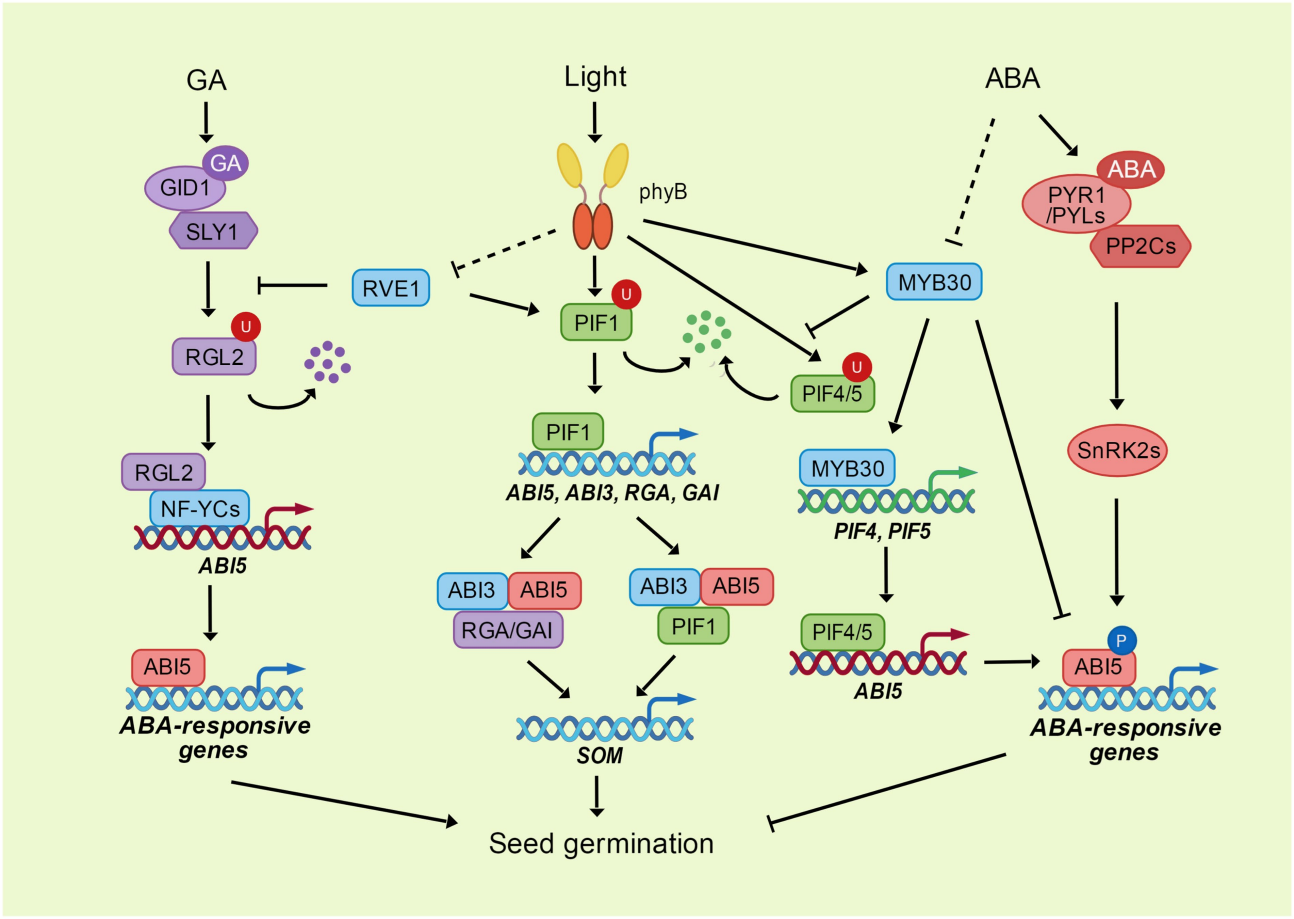
The phyB-PIFs module integrates the light, ABA and GA signaling in seed germination. ABI5 inhibits seed germination through the promotion of ABA-responsive genes. ABA activates the transcriptional activity of ABI5 through the phosphorylation mediated by SnRK2s. MYB30 directly interacts with ABI5 to repress its transcriptional activity. DELLA protein RGL2 interacts with NF-YC transcription factors to enhance the expression of *ABI5* through direct binding to *ABI5* promoter. GA promotes the degradation of RGL2 through its receptor GID1 and ubiquitin E3 ligase SLY1. The photoreceptor phyB perceives and transduces the light signal through its downstream transcription factors. PhyB interacts with PIF transcription factors to promote their degradation through ubiquitination. PIF1 directly enhances the expression of *ABI3*, *ABI5*, *RGA*, and *GAI*. ABI3 acts as a activator of ABI5 by its interaction with ABI5. RGA, GAI and PIF1 can interact with both ABI3 and ABI5 to coregulate the expression of *SOM*, which controls the GA and ABA metabolism. PhyB interacts with MYB30 and enhances the expression and stability of MYB30 under light, while MYB30 directly activates the expression of *PIF4* and *PIF5*, and increases their stability through repressing the interaction between PIF4/5 and phyB. PIF4 and PIF5 bind to *ABI5* promoter and enhance *ABI5* expression to activate the ABA signaling in the dark. Another MYB transcription factor, RVE1, mediates the light signaling transduce of phyB. RVE1 interacts with PIF1 and enhances its binding to *ABI3* promoter. RVE1 also interacts with RGL2 and protects RGL2 from degradation through inhibiting the interaction between RGL2 and SLY1.

RGL2 has been identified as a core negative regulator of seed germination. As mutation of RGL2 can rescue the germination phenotype of *ga1* without GA application under light conditions (Lee et al., 2002; Tyler et al., 2004). Other DELLA proteins, including GA insensitive (GAI), RGA and RGL1, also participate in seed germination regulation by enhancing the function of RGL2 (Cao et al., 2005; Piskurewicz et al., 2009). RGL2 functions as an important hub for the crosstalk between GA and ABA signaling, as the function of RGL2 in seed germination depends on ABI5 (Figure 1). The expression and stability of ABI5 were dramatically induced under low-GA conditions, but all these effects were repressed in the *rgl2* mutant. Overexpression of ABI5 restores the insensitivity of *rgl2* to PAC treatment (Piskurewicz et al., 2008), demonstrating that ABI5 functions genetically downstream of RGL2 in controlling seed germination. Indeed, RGL2 can directly bind to the promoter of *ABI5* to promote its expression, and the interaction between RGL2 and NUCLEAR FACTOR-Y C (NF-YC) proteins enhances their ability to activate *ABI5* expression (Liu et al., 2016). In addition, the DELLA protein GAI and RGA physically interact with ABI5 to enhance the expression of transcription factor *SOMNUS* (*SOM*), which activates the genes responsible for ABA biosynthesis but represses that for GA biosynthesis (Kim et al., 2008; Lim et al., 2013).

DELLA proteins interact with different transcription factors to activate or inhibit their transcriptional activity, thereby controlling a variety of plant development processes (Claeys et al., 2014). However, it is unclear whether DELLA proteins affect the transcriptional activity of ABI5, although both of them can activate the expression of *SOM* and repress the expression of *GASA6* (Lim et al., 2013; Zhong et al., 2015). In addition, how DELLA proteins regulate the stability of ABI5 is still unclear. In germinated seeds of the *sly1* mutant, which is responsible for degrading DELLA proteins, although the protein content of RGL2 remained high, the ABI5 protein decreased significantly with germination time, indicating that there is a more complex mechanism for the regulation of ABI5 stability by RGL2 (Piskurewicz et al., 2008).

In contrast to GA, ABA stimulates *RGL2* expression and enhances its stability. The *rgl2* mutant also shows insensitivity to ABA, which can be recovered by the expression of *ABI5* (Piskurewicz et al., 2008; Liu et al., 2016). However, the underlying mechanism by which ABA regulates RGL2 to antagonize GA signaling remains largely elusive. In the ABA signaling pathway, the ABA receptor PYLs affects the stability of ABI5, while ABI5 positively regulates the expression of *PYLs* (Zhao et al., 2020). Considering that ABA also dramatically enhances the expression and stability of ABI5 and that RGL2 has a close connection with ABI5, the regulation of RGL2 by ABA may be related to ABI5. At the protein level, as the major regulator of RGL2 protein stability, SLY1 is a good candidate for this purpose. As SLY1 is also involved in ABA-mediated seed germination repression, *sly1* mutant seeds show increased sensitivity to ABA (Strader et al., 2004). Studying how ABA regulates RGL2 will help us to understand the balance of GA and ABA signaling during seed germination.

## Light integrates ABA and GA signaling during seed germination

Light is a primary environmental cue that guides seed germination (Kami et al., 2010; Li et al., 2011). In Arabidopsis, far-red light inhibits seed germination, whereas red light promotes seed germination. The phytochrome photoreceptors that absorb red/far-red light are essential for triggering light-mediated seed germination. Among the five phytochromes, phytochrome B (phyB) plays a dominant role in the initial phase of seed germination, and mutation of phyB prevents seeds from germinating under red light (Shinomura et al., 1994, 1996; Seo et al., 2009). Upon far-red light exposure, phytochromes become inactivated (Pr) and are located in the cytosol. After absorbing red light, phytochromes are converted to their activated (Pfr) forms and translocate into the nucleus, where they transduce light signaling by interacting with many transcription factors (Li et al., 2011).

### PIFs: The communication hub for light, ABA, and GA signaling

The bHLH transcription factors PHYTOCHROME-INTERACTING FACTORs (PIFs), which can directly interact with phyB (Leivar and Quail, 2011), play crucial roles in repressing photomorphogenesis and seed germination (Figure 1). PIF1, also known as PIF3-LIKE5 (PIL5), is a key negative regulator of phytochrome-mediated seed germination. Overexpression of PIF1 represses germination under red light, and mutation of PIF1 can partially rescue germination inhibition in the *phyB* mutant (Oh et al., 2004). Red light activates phyB to promote the degradation of PIF1 (Oh et al., 2006; De Wit et al., 2016). PIF1 indirectly regulates the expression of GA and ABA biosynthesis and catabolic genes *via* the activation of DOF AFFECTING GERMINATION 1 (DAG1) and SOM (Kim et al., 2008; Oh et al., 2009; Gabriele et al., 2010). In contrast, PIF1 directly binds to the promoters of *RGA* and *GAI* to promote their transcription and repress GA signaling (Oh et al., 2007). Moreover, PIF1 induces the expression of *ABI3* and *ABI5* to stimulate ABA signaling, and PIF1 can directly bind to the G-box motifs in the ABI5 promoter to activate the transcription of ABI5 under ABA treatment in the dark (Oh et al., 2009; Qi et al., 2020). On the other hand, ABI5 can directly interact with PIF1 to enhance the binding ability of PIF1 to its targets, and ABI3, the enhancer of ABI5, interacts with PIF1 to collaboratively activate the expression of *SOM* (Park et al., 2011; Kim et al., 2016). AP2/ERF transcription factors ERF55 and ERF58 directly bind to the promoters of *PIF1*, *SOM* and *ABI5*, and induced their expression. While phyA and phyB interact with ERF55/58 to inhibit their DNA-binding activity, thus modulating light-mediated regulation of germination (Li et al., 2022).

In addition to light-associated seed germination, PIFs, including PIF1, PIF3, PIF4, and PIF5, also participate in ABA signaling by regulating the expression of *ABI5* in the dark (Qi et al., 2020). The quadruple mutant *pifq* (*pif1 pif3 pif4 pif5*) seeds show significant insensitivity to ABA during seed germination. In addition to PIF1, PIF3, PIF4 and PIF5 also activate the transcription of *ABI5* by directly binding to its promoter (Qi et al., 2020). Additionally, PIFs can physically interact with the ABA receptors PYL8 and PYL9, and this interaction promotes PIF4 protein accumulation in the dark and enhances PIF4 binding to the *ABI5* promoter but negatively regulates PIF4-mediated ABI5 activation (Qi et al., 2020). Considering that ABI5 can directly enhance the expression of *PYLs* during seed germination (Zhao et al., 2020), PYLs, PIFs and ABI5 seem to form a regulatory loop to modulate the ABA signaling in seed germination.

PIF3 and PIF4 are also important hubs for the connection between light and GA signals. DELLA proteins interact with PIF3/4 and block their binding to target genes to regulate photomorphogenesis (de Lucas et al., 2008; Feng et al., 2008). In the absence of GA, DELLA proteins interact with PIF3 and repress its DNA-binding activity, thus blocking PIF3-mediated hypocotyl elongation. In the presence of GA, GID1 proteins promote ubiquitination and proteasome-mediated degradation of DELLA proteins, therefore releasing PIF3 from the repression of DELLA proteins (Feng et al., 2008). Considering the functions of DELLA proteins, PIF3 and PIF4 in seed germination, the DELLAs-PIFs module may also be an important hub for the coordinated regulation of light, GA, and ABA signaling.

### MYB transcription factors: The connection between phyB and its downstream response

In addition to PIFs, phyB also transduces light signaling through other transcription factors. Among them, MYB transcription factors play an important role in the integration of different signals (Figure 1). REVEILLE1 (RVE1) and RVE2, two MYB-like transcription factors, suppress phyB-mediated seed germination by directly controlling GA biosynthesis and catabolism (Jiang et al., 2016). RVE1 physically interacts with PIF1 and their interaction enhances the abilities of PIF1 to activate *ABI3* and RVE1 to repress *GA3ox2.* PIF1 and RVE1 directly bind to each other’s promoters to activate expression, thus forming a transcriptional feedback loop to coordinately inhibit seed germination (Yang et al., 2020a). In addition, RVE1-mediated inhibition of seed germination is dependent on the DELLA protein RGL2. RVE1 interacts with RGL2 and protects RGL2 from proteasome-mediated degradation by repressing the interaction between RGL2 and its ubiquitin E3 ligase SLY1 (Yang et al., 2020b).

MYB30, another MYB transcription factor, interacts with phyA and phyB and mediates their function in photomorphogenesis. phyA and phyB induce the expression of *MYB30* under light conditions, and mutation of MYB30 can repress the long hypocotyl phenotypes of *phyA* and *phyB* mutants under FR and R/W light. The function of MYB30 in photomorphogenesis is dependent on PIF4 and PIF5 proteins. MYB30 directly induces *PIF4* and *PIF5* expression by binding to their promoters, and MYB30 enhances their stability by inhibiting the interaction of PIF4 and PIF5 with phyB. Furthermore, MYB30 physically interacts with PIF4 and PIF5, thus acting additively to repress photomorphogenesis (Yan et al., 2020). In addition to its function in photomorphogenesis, MYB30 is also a critical repressor of ABA-mediated seed germination (Zheng et al., 2012). MYB30 interacts with ABI5 and interferes with its transcriptional activity to restrict the function of ABI5 during seed germination (Nie et al., 2022). The ubiquitin E3 ligase MIEL1 interacts with and ubiquitinates MYB30 and ABI5, thus mediating the turnover of MYB30 and ABI5 during seed germination. ABA represses the function of MIEL1 by inducing its degradation and repressing its interaction with MYB30 and ABI5. The MIEL1-MYB30 repression module precisely regulates ABI5, thus ensuring the restriction of ABA signaling during seed germination (Nie et al., 2022).

### COP1: The regulator that controls ABI5 expression and stability

The E3 ubiquitin ligase CONSTITUTIVELY PHOTOMORPHOGENIC1 (COP1) functions as a central switch in light signaling and links photoreceptors with downstream factors to coordinate the light response (Lau and Deng, 2012; Han et al., 2020). COP1 interacts with SUPPRESSOR OF PHYAs (SPAs) to promote the degradation of positive regulators of light signaling by the 26S proteasome (Hoecker, 2017). The substrates of the COP1/SPA E3 ligase mainly include phyA and phyB (Seo et al., 2004; Jang et al., 2010), the critical photomorphogenesis-promoting transcription factor ELONGATED HYPOCOTYL5 (HY5; Osterlund et al., 2000) and its homolog HYH (Holm et al., 2002), and a series of B-BOX (BBX) proteins (Xu et al., 2016; Lin et al., 2018). COP1 localizes to the nucleus in the dark; upon light exposure, COP1 migrates from the nucleus to the cytosol, thus allowing the nuclear accumulation of photomorphogenesis-promoting factors (such as HY5) and the initiation of photomorphogenesis (Lau and Deng, 2012; Hoecker, 2017; Han et al., 2020).

**Figure 2:**
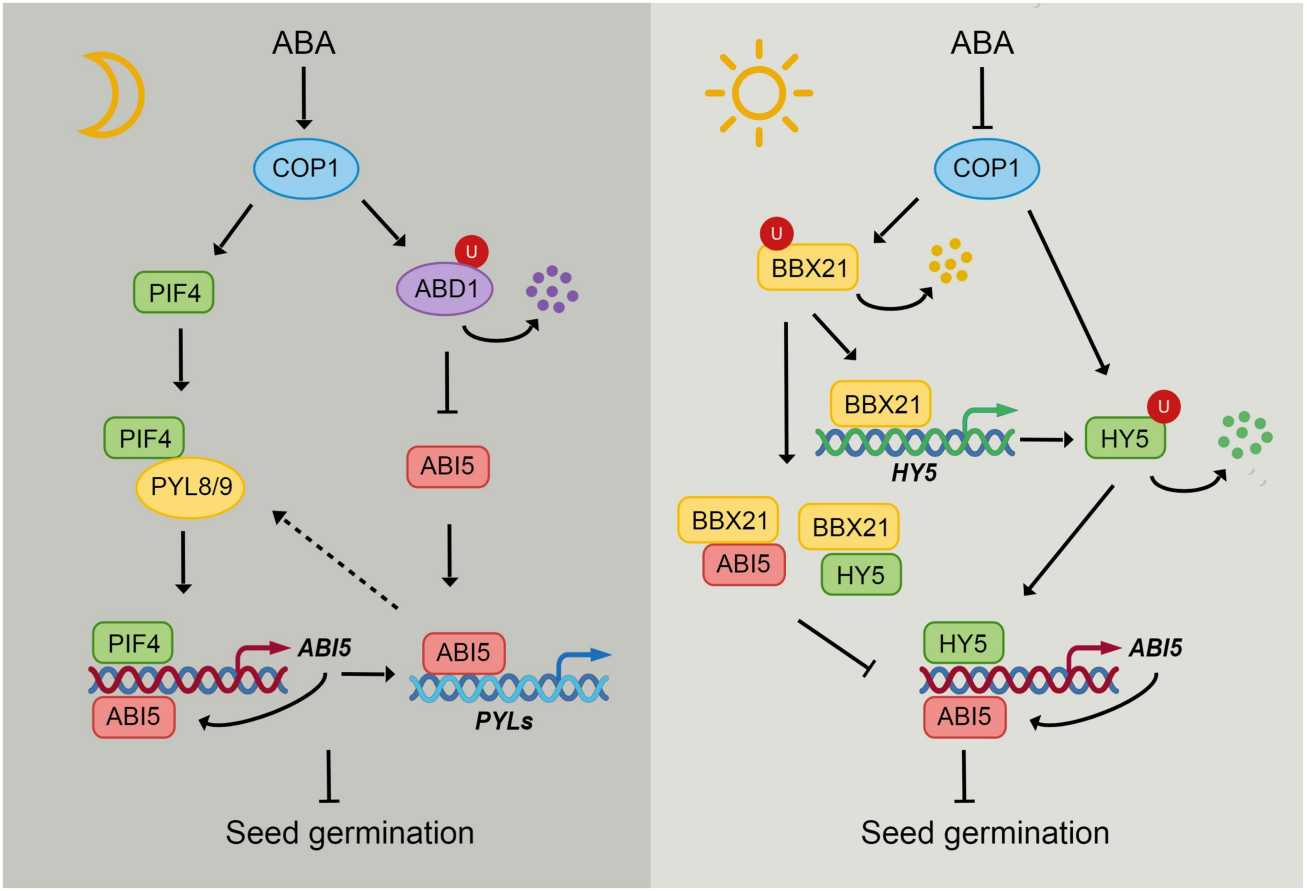
The integration of light and ABA signaling through COP1. In the dark, COP1 localizes in nucleus and ABA induces the nuclear accumulation of COP1 in darkness. COP1 enhances the stability of PIFs, thus leading to the activation of *ABI5* expression by PIFs. ABA receptors PYL8 and PYL9 interact with PIF4 to enhance its stability but repress its transcriptional activity. ABI5 enhances its own expression by directly binding to its promoter. ABI5 also activates the expression of *PYLs* during seed germination, which may in turn regulate the function of PIFs. During seedling growth in darkness, COP1 protects ABI5 from degradation by targeting ABD1 for 26S proteasome-mediated degradation. COP1 interacts with transcription factors HY5 and BBX21 and promotes their proteasome-mediated degradation. Light and ABA promote COP1 export from the nucleus, thus inhibiting its function in nucleus. HY5 directly activates the expression of *ABI5*. While BBX21 interacts with HY5 and ABI5 to repress their ability of binding to *ABI5* promoter. However, BBX21 enhances *HY5* expression by directly binding to *HY5* promoter.

In addition to its pivotal role in regulating seedling photomorphogenesis, COP1 is also involved in seed germination and seedling growth, which is closely linked to the ABI5 protein (Figure 2). During postgermination stage, COP1 facilitates the binding of ABI5 to its target promoters to arrest seedling development (Yadukrishnan et al., 2020). In addition, COP1 positively regulates ABA-inhibited seedling growth by enhancing the stability of ABI5 in the dark. COP1 physically interacts with ABD1, a substrate receptor of the CUL4-based E3 ligase responsible for ABI5 degradation, and ubiquitinates ABD1 to facilitate its degradation. ABA induces nuclear accumulation of COP1 in the dark, thus activating COP1-mediated ABD1 degradation and ABI5 protein accumulation (Peng et al., 2022). In contrast, COP1 negatively regulates ABA-mediated seed germination inhibition, as the *cop1* mutant shows hypersensitivity to ABA under both light and dark conditions (Chen et al., 2022; Peng et al., 2022). Under light conditions, ABA promotes COP1 enrichment in the cytoplasm, leading to the accumulation of HY5 and ABI5 proteins in the nucleus and increased ROS levels in seeds (Chen et al., 2022). But how COP1 regulates ABI5 during seed germination under dark is still unclear.

The transcription factor HY5 is also involved in the ABA response. The *hy5* mutants are hyposensitive to ABA in terms of seed germination, postgermination growth and plant lateral root growth. HY5 can directly bind to the *ABI5* promoter to activate the expression of *ABI5* (Chen et al., 2008). The B-box protein BBX21, a positive regulator of seedling photomorphogenesis, negatively regulates ABA signaling by interacting with HY5 and ABI5 to interfere with their binding to the ABI5 promoter (Xu et al., 2014a). In addition, BBX21 recruits the chromatin remodeler protein HRB2 to the *ABI5* promoter to repress ABI5 expression (Kang et al., 2018). In contrast, BBX19 suppresses seed germination by activating *ABI5* transcription (Bai et al., 2019). It is worth noting that HY5 and BBX21 are the direct substrates of COP1. COP1 targets both HY5 and BBX21 for 26S proteasome-mediated degradation (Xu et al., 2016). COP1 may also participate in the ABI5-mediated ABA response process by regulating HY5 and BBX21.

PIFs are also important for the connection between COP1 and ABI5, as COP1 positively regulates PIF protein levels in the dark. Overexpression of PIF1, PIF3, PIF4 and PIF5 suppresses *cop1* phenotypes in the dark (Pham et al., 2018a). The COP1-SPA complex enhances PIF3 stability by repressing the kinase activity of BIN2, which induces PIF3 degradation *via* the 26S proteasome (Ling et al., 2017). COP1-SPA stabilizes PIF5 in the dark but promotes the degradation of PIF5 in response to red light through ubiquitination (Pham et al., 2018b). In addition, SPA1 can directly interact with and phosphorylate PIF1, leading to the ubiquitination and degradation of PIF1. PhyB interacts with SPA1 and enhances the recruitment of PIF1 for phosphorylation (Paik et al., 2019). Considering the activation of ABI5 by PIFs, COP1 may also participate in seed germination by PIF-mediated ABI5 regulation.

### Other light factors that regulate ABI5

Moreover, a variety of components that regulate light signals have been identified to participate in ABA signaling through the regulation of ABI5. As positive regulators of far-red signaling, the transcription factors ELONGATED HYPOCOTYL3 (FHY3) and FAR-RED IMPAIRED RESPONSE1 (FAR1) can activate *ABI5* expression by directly binding to its promoter (Tang et al., 2013). The light-mediated development protein DET1 physically interacts with FHY3 and represses FHY3-mediated activation of *ABI5*. DET1 physically interacts with histone deacetylase HDA6 and recruits HDA6 to the *ABI5* promoter, leading to the repression of *ABI5* expression by the enrichment of H3K27ac and H3K4me3 modifications (Xu et al., 2020). In addition, DET1 can enhance the stability of PIF1 and PIF4, which can activate the expression of *ABI5* (Shi et al., 2015; Gangappa and Kumar, 2017). However, DET1 interacts with COP1 and promotes COP1-mediated degradation of HY5, which acts as a positive regulator of *ABI5* expression, suggesting a more complicated role of DET1 in regulating ABI5 (Cañibano et al., 2021).

## Concluding remarks

Seed germination is one of most important physiological processes, which determines the time when the plant starts to grow. Since plant growth is severely restricted by the environment, the precise regulation of germination helps plants grow in a more favorable environment. In the last two decades, many new advances have been made in elucidating the key components in seed germination control. Although the regulation of seed germination is a very complex process, there is increasing evidence that ABI5 can act as a final inhibitor of various signals regulating seed germination, including not only ABA, GA, and light signals but also auxin, BR and JA and other signals (Skubacz et al., 2016). In this paper, we present the current understanding of ABA, GA, and light signaling networks in regulating seed germination through the regulation of the important hub, ABI5. At the same time, DELLA proteins, PIF transcription factors and COP1 E3 ligase also play critical roles in this signaling crosstalk. Although the regulatory mechanisms of seed germination have been widely studied, there are still problems to be solved. First, how is GA and ABA signaling balanced by the DELLAs-ABI5 module? RGA and GAI can interact with ABI5, but whether RGL2 can participate in the regulation of seed germination through direct interaction with ABI5, similar to RGA and GAI, is still unknown. How ABA regulates DELLA proteins, especially RGL2, to inhibit GA signaling also needs to be further investigated. The regulation of ABI5 under the crosstalk of GA and ABA seems to be a key to unlock these questions. Second, both PIF and DELLA proteins can regulate the expression of *ABI5*, and they can directly physically interact each other in the regulation of photomorphogenesis, but it is not known whether they coordinately regulate seed germination, especially through the control of *ABI5* expression. COP1 can affect the stability of both DELLA and PIF proteins, but it is still unclear whether COP1 participates in seed germination through the regulation of their functions. The function of these core light signaling components in seed germination still needs to be integrated. Finally, the downstream targets of ABI5 are still poorly understood, the revealing of ABI5 function is very important for our comprehensive understanding of the regulatory mechanism of seed germination.

## Author contributions

HZ and YZha wrote the manuscript and prepared the figures. YZhe contributed to the conception and interpretation of data, revision of the manuscript content, and final approval. All authors contributed to the article and approved the submitted version.

## Funding

This work was supported by the National Natural Science Foundation of China (31872656); the Natural Science Foundation of Henan (212300410022) and the Program for Innovative Research Team (in Science and Technology) in University of Henan Province (21IRTSTHN019).

## Conflict of interest

The authors declare that the research was conducted in the absence of any commercial or financial relationships that could be construed as a potential conflict of interest.

## Publisher’s note

All claims expressed in this article are solely those of the authors and do not necessarily represent those of their affiliated organizations, or those of the publisher, the editors and the reviewers. Any product that may be evaluated in this article, or claim that may be made by its manufacturer, is not guaranteed or endorsed by the publisher.
